# Towards an integrated model for supervision for mental health and psychosocial support in humanitarian emergencies: A qualitative study

**DOI:** 10.1371/journal.pone.0256077

**Published:** 2021-10-06

**Authors:** Camila Perera, Kelly A. McBride, Áine Travers, Pia Tingsted Blum, Nana Wiedemann, Cecilie Dinesen, Byron Bitanihirwe, Frédérique Vallières

**Affiliations:** 1 International Federation of the Red Cross and Red Crescent Societies Reference Centre for Psychosocial Support, Copenhagen, Denmark; 2 Trinity Centre for Global Health, School of Psychology, Trinity College Dublin, The University of Dublin, College Green, Dublin, Ireland; South African Medical Research Council, SOUTH AFRICA

## Abstract

**Background:**

Despite recent advances in the development and provision of mental health and psychosocial support (MHPSS) in humanitarian settings, inadequate supervision remains a significant barrier to successful implementation. The present study sought to incorporate broad stakeholder engagement as part of the first phase of development of a new Integrated Model for Supervision (IMS) for use within MHPSS and protection services in humanitarian emergencies.

**Methods:**

Semi-structured interviews were conducted with 26 global mental health professionals. Data was analysed thematically, using a combination of inductive and deductive methods. Codes and themes were validated through co-author cross-checks and through a webinar with an expert advisory group.

**Results:**

Results reinforce the importance of effective supervision to enhance the quality of interventions and to protect supervisees’ wellbeing. Participants generally agreed that regular, supportive supervision on a one-to-one basis and as a separate system from line management, is the ideal format. The interviews highlight a need for guidance in specific areas, such as monitoring and evaluation, and navigating power imbalances in the supervisory relationship. Several approaches to supervision were described, including some solutions for use in low-resource situations, such as group, peer-to-peer or remote supervision.

**Conclusion:**

An integrated model for supervision (IMS) should offer a unified framework encompassing a definition of supervision, consolidation of best practice, and goals and guidance for the supervisory process.

## Introduction

Populations affected by humanitarian emergencies are exposed to several stressors, including violence, lack of safety, loss of livelihoods and disruption of social networks. Such adversity is associated with significant emotional distress (e.g., feelings of anxiety and sadness, hopelessness, difficulty sleeping, fatigue, irritability or anger and/or aches and pain) [1]. Emergencies can also induce or exacerbate more complex mental health problems, with approximately one-in-five people in post-conflict settings experiencing clinical depression, anxiety disorder, post-traumatic stress disorder, bipolar disorder, or schizophrenia [2]. This prevalence is significantly higher than that reported by the World Mental Health survey analysis of general population samples from 14 countries, where the prevalence of serious disorders was estimated at 0.4%-7.7% [3].

Humanitarian crises are most likely to occur in low and middle-income countries (LMICs) [4], where human resources for mental health are scarcest. Data from 2014–2016 indicates that low-income countries have 0.1 psychiatrists and 0.3 psychiatric nurses per 100 000 people [5]. The availability in high income countries is 120 times greater for psychiatrists and 75 times greater for psychiatric nurses. The composite term ‘Mental Health and Psychosocial Support’ in humanitarian settings describes “any type of local or outside support that aims to protect or promote psychosocial wellbeing and/or prevent or treat mental disorders”[6]. Because of shortages of trained personnel, many MHPSS programmes are based on a task-shifting or task-sharing approach [7], whereby lay providers are trained to deliver services and interventions, under the supervision of more specialist cadres (i.e. psychotherapists, clinicians, and qualified counsellors). Effective supervision is thus an essential component of MHPSS programming.

The importance of supervision in MHPSS programming is recognised across various guidelines [6, 8, 9]. This recognition is consistent with decades of research within broader global health programming, which highlights supervision as a key determinant of health worker performance, satisfaction, wellbeing, commitment, motivation, and retention [10–13]. In addition to the benefit of supervision for practitioners, research suggests that supervision may also be an important determinant of client outcomes. One study in a specialised high-income setting found that supervision accounted for 16% of variance in client outcomes [14]. Similarly, a meta-analysis of client outcomes relating to depression found regular supervision to be significantly associated with study effect sizes [15].

However, despite this widespread recognition of its importance, supervision is frequently neglected in community health programming, including MHPSS programmes [16, 17]. There are various factors that may contribute to this programmatic oversight, including lack of funding, resistance by higher cadres, hierarchal organisational structures and language barriers, as well as a lack of clear guidance on what supervision is and how it should be conducted [17]. Additionally, research focusing specifically on delivery of MHPSS supervision in humanitarian emergencies is scarce, and given the particular stressors and difficulties associated with delivering MHPSS interventions in such settings [18, 19] it is suggested that specific consideration for humanitarian contexts is warranted. The current study sought to address these gaps by incorporating global stakeholder engagement in the first phase of development of the Integrated Model for Supervision (IMS) for specific use within MHPSS and protection services in humanitarian emergencies. The IMS and accompanying materials (e.g., cultural adaptation guidance, monitoring and evaluation tools and training materials) will be aligned with the IASC Guidelines on MHPSS in Emergency Settings [6], thereby contributing to establishing and raising the quality of routine supervision in MHPSS globally. The model will provide comprehensive guidance not only to supervisors on key skills needed in order to carry out the role effectively, but also to organisations to adapt their workflows and contribute to addressing the higher-level barriers to implementation As part of this formative phase of the IMS, the current study had three objectives:

1. To define what is commonly understood as ‘supervision’ for MHPSS programming in humanitarian emergencies
2. To identify best practice for supervision within MHPSS programming
3. To identify how supervision can be practically incorporated into existing MHPSS programming

## Materials and methods

### Study design

The study objectives were addressed using a qualitative design, with interview protocols informed by the results of a rapid desk review [20]. Ethical approval for the study was obtained from the Centre for Health Policy and Management/Centre for Global Health Research Ethics Committee of Trinity College Dublin (Dublin, Ireland) on January 17^th^, 2020.

### Participants and procedures

Semi-structured interviews were conducted by the second author (KM; female), a licensed mental health professional and technical expert in MHPSS, employed by the IFRC Psychosocial Centre, and experienced in conducting training and supervision in MHPSS. A total of 26 stakeholders (19 female, 7 male), were interviewed, including both supervisors and supervisees. The interviewer had prior professional relationships with some interviewees. Professional categories that were represented included: technical experts (*n* = 18), representatives from health ministries (*n* = 2), academics (*n* = 1), volunteers (*n* = 1) and donors (*n* = 4). A technical expert was defined as anyone providing specific knowledge or expertise to MHPSS and protection teams, either in headquarters or through the field offices of the organisation. These individuals represented 23 non-governmental and governmental institutions providing MHPSS and protection services in humanitarian and low-resource settings. The majority of the sample (*n* = 20) were employed by non-governmental humanitarian agencies, many in senior roles with regional or global responsibilities (*n* = 13). Sampling of respondents for the qualitative interviews was purposive, followed by snowball sampling. Purposive sampling was based on the results of a stakeholder mapping exercise, which aimed to incorporate a range of professional perspectives from the field of MHPSS. Snowball sampling was conducted through purposively sampled participants by asking whether they could recommend other individuals with expertise in MHPSS who could offer insight relevant to the study objectives. Initial contact was made with all participants via email. Following a positive response, the interviewer arranged a date and time for the interview to take place, and sent a Zoom link and calendar invite to the participant. A total of 68 stakeholders were invited to participate. Those who did not participate either declined participation or did not respond, with some participants contacted in the snowball sampling procedure responding after the deadline had passed. A total of 27 individuals completed interviews; one participant completed an interview but chose to withdraw their data due to concerns about the professional sensitivity of its content.

### Tools

A qualitative interview guide (S1 File) was developed based on the outcomes of the aforementioned desk review of peer-reviewed and grey literature on MHPSS supervision. The interview guide was reviewed by the project advisory group, and two pilot interviews were conducted. Questions were designed to elicit interviewees’ reflections on each of the three study objectives. To address Objective 1, participants were asked to reflect on their experience of supervision within MHPSS programming and their understanding of a supervisor’s role. To address Objective 2, participants were invited to discuss the factors that make for a good supervisory relationship. In the instance where informants described their experience as a supervisee, questions were asked about supervision frequency and helpful or unhelpful practices within sessions. Where informants described their experience as a supervisor, questions were asked about the training, if any, they had received in preparation for the role, different formats of supervision they had engaged with, and the elements they felt contributed to its successes and challenges. Finally, to address Objective 3, informants were asked about their experience of how supervision is incorporated practically within MHPSS programming at organisational or sector-wide level, and any gaps that currently exist in MHPSS supervision.

### Data collection and analysis

Interviews were conducted in English between February 14^th^ and May 6^th^, 2020. Participants were interviewed online via Zoom and each interview lasted approximately one hour. Only the interviewee and interviewer were present on the Zoom calls. At recruitment stage, and again at the beginning of the interviews, participants were informed that the interview data would inform the development of a new integrated model for the supervision of MHPSS activities in humanitarian emergency settings. Participants were made aware that the project was a collaboration between Trinity Centre for Global Health and the IFRC, and that the interviewer was contracted to work on developing the new supervision model. Each participant was interviewed only once, and advised that they may contact the interviewer to make any additional comments of clarifications by email if they wished. However, no further data was submitted in this way.

Interviews were audio recorded, with informed verbal consent obtained from participants. The interviewer took field notes which were de-identified and securely stored to assist thematic development. Recordings were transcribed verbatim and transcripts were de-identified by removing names, affiliation, and locations. A random sample of transcripts were checked for accuracy against recordings. Participants were offered the opportunity to view their transcripts and redact or change any information prior to publication and were asked to verify and approve direct quotes prior to inclusion in the study. All the quotes proposed were approved to be included, with only minor grammatical and syntactical changes requested. Data was analysed thematically in NVivo 12, using a combination of inductive and deductive methods [21]. First, an *a priori* coding framework was developed based on the findings from the rapid desk review. New codes were then added to reflect additional insights emerging from the interviews. A coding tree was developed by the first author (CP) and discussed with the last author (FV). Following this, the codes were revised to identify common themes in the data. Finally, the identified themes were grouped under their corresponding objectives, as presented below. The coding and theme development process was completed by two authors (CP and BB) and further checked and validated by the third author (ÁT). The themes were refined in collaborative discussions among all co-authors, before presentation and further validation through a webinar with an advisory group responsible for overseeing the development of the IMS. The webinar included a presentation of the interview findings, followed by a critical discussion to aid further synthesis.

## Results

### Objective 1: Defining supervision

#### Supervision is essential to sustainable capacity building and quality of care

Participants felt strongly that supervision should be a prerequisite of MHPSS service provision. Informants described supervision as a safe, supportive, confidential and collaborative meeting between a supervisor and supervisee, where supervisees can reflect on their difficulties and mistakes, be reinforced for their successes, receive constructive criticism and build their technical skills and capacity.

In humanitarian settings, supervisees’ knowledge of mental health, especially at early stages of their practice, often relies on skills acquired through short-term, intense trainings, usually provided by external experts. Although such trainings provide a thorough introduction to relevant concepts and interventions, MHPSS providers nonetheless encounter challenges in their practice for which they need additional support. Supervision thus plays a fundamental role in MHPSS providers’ professional development by strengthening and consolidating the knowledge gained through training. As explained by Participant E:

*Real training*, *of course*, *requires supervision*. *This [supervision] is what changes behaviour and builds skills*. *Without supervision*, *you cannot build skills*. *After the workshop people of course gain knowledge and perhaps their attitudes change but to build skills you need supervision*.

(Academic)

Supervision also helps supervisees to manage their insecurities and challenge any prejudices or biases that may affect their professional judgment. As explained by Participant N:

*I think that a supervisor should be willing and able to challenge the supervisee to recognise dynamics in their relationship with the client that might not be helpful*…*really being able to help the supervisee reflect on themselves*.

(Technical Expert—Field)

Supervision should ensure that supervisees’ workload is manageable and considerate of the difficulty of cases, programmes and operational environment. The supervisory process should function to empower supervisees to ethically and confidently provide MHPSS, as well as conduct referrals as necessary. As such, supervisors’ primary purpose is, as described by Participant G, to act as *“guardians of quality”*, safeguarding the well-being of both supervisees and beneficiaries.

Insufficient supervision was seen to as a risk to service quality, affecting the wellbeing of beneficiaries and supervisees alike. Risk to supervisees was considered particularly important when delivering MHPSS through lay providers:

*Organisations that utilise para-professional staff*, *who don*’*t necessarily have formal qualifications related to mental health*, *are doing them and the community a disservice by not providing adequate supervision*. *Allowing staff to practice without supervision places too much responsibility on the para-professional staff which puts them at risk of burnout and stress and puts their own well-being at risk*.

(Participant A, Technical Expert—HQ)

#### The supervisory role is often overlooked, unrecognised and poorly defined

Despite its noted importance, and although some reported that their organisations had worked to prioritise supervision in recent years, several participants expressed the view that the supervisory role is commonly overlooked and poorly defined. Some informants described times when the support they required was completely unavailable:

*When I was back from a mission*, *I always went to see a psychologist myself*, *but [this was] my own initiative*, *my own money*. *And when I was working for [organisation] that wasn*’*t easy at all*…*it was very expensive*…*it was difficult*.

(Participant G, Technical Expert—HQ)

The main reason cited for under-prioritisation was lack of funding, and even those who felt positively about their organisation’s prioritisation of supervision reported that the area is generally under-resourced. Another issue raised by participants was that specialists (e.g. MHPSS technical advisors, psychiatrists, psychologists) are often requested to supervise in addition to their full-time role (e.g., programme design, implementation, evaluation and proposal writing). Often, they are requested to do so without guidance on how to supervise, with little knowledge of the needs or capacity of supervisees, without supervision being a clear component of their job descriptions and irrespective of whether they have previous experience in providing or receiving supervision. Participants felt that organisations often do not consider supervision as an essential function. As a result, specialists may find themselves in a role that they did not apply for, are not motivated to do, and may not feel qualified for or have time to fulfil.

#### Supervision should include (but should not only consist of) caring for staff well-being

Interviewees agreed that supervisees’ well-being should be an important focus of supervision. While a few organisations have dedicated personnel whose sole responsibility centres on staff care and well-being, most assign this responsibility to the supervisor. Most participants agreed that staff and volunteer care is a highly neglected area and that supervisors are ideally placed to monitor, prevent, identify, and address difficulties faced by supervisees. However, while staff wellbeing emerged as an important aspect of supervision, some participants warned against this becoming the sole objective of supervision and the sole responsibility of supervisors, where they are available. Participants felt that supervisors should monitor for signs of significant stress or faltering motivation and have avenues to refer their supervisees to staff support. The importance of the staff care component of supervision was outlined by Participant N:

*One of the things that actually helped me from the Narrative Therapy framework is the saying that we have is* ’*the personal is professional*’. *So*, *whatever we have going on personally will influence us professionally*…*S/ o just having a recognition of that was really helpful for me to be able to talk more openly with my supervisees about like what is going on for them and how that might affect their work with their client and for you to be like we are all humans and have something going on*.

(Technical Expert—Field)

### Objective 2: Identifying best practice

#### Supervisors should be experienced and approachable, with strong communication skills and understanding of the cultural and social context

Informants identified several attributes of a good supervisor, including supportiveness, trustworthiness, patience, calmness, approachability and empathy. Participants felt that good supervisors are passionate about strengthening supervisees’ skills and stimulating supervisees’ thinking. They possess strong facilitation skills, as well as good conflict resolution, coaching and communication skills. The types of actions described as reflecting those qualities include active listening, asking open questions that encourage reflection, communicating clearly and calmly to create a safe space. Some examples of reflective questioning described by Participant A (Technical Expert) included: Now you have a bit more insight what would you change? What wouldn’t you change? What challenges have you had and how have you tried to tackle those challenges? What is going well and why do you think it is going well?

Characteristics of good supervisors are further discussed by participants L and D (Technical Experts):

*A good supervisor is someone who is calm*, *of course*, *calmness is at the heart of everything*…. *They have to be*…*really knowledgeable and highly experienced*… *someone who is unshakable*, *who doesn*’*t get wound up too quickly or panic*. *They should be able to be good containers*, *generous and humble*, *not grand-standers*. *They shouldn*’*t want or need to be at the centre of attention*. *They need good facilitation skills and be able to connect with people from lots of different backgrounds*, *abilities*, *identities and contexts*…

(Technical Expert, HQ)

…*approachability and feeling that they [supervisees] can be vulnerable and say* ’*I don*’*t know what to do*’ *without feeling like they are going to be ridiculed for not knowing*…*so being approachable and being a kind and listening ear for them*…

(Technical Expert—Field)

Informants also expressed that supervisors should value supervisees’ opinions, skills, and understanding of the beneficiaries’ needs. They should be appreciative of supervisees’ efforts, convey interest in their work and offer new insights, alternative approaches or innovations. Supervisors should be professional and responsive to supervisees’ needs and learning styles, and they should respect the boundaries of the supervisor-supervisee relationship. This can be achieved, for example, through open discussion of both supervisors’ and supervisees’ needs and expectations from sessions.

Key informants varied somewhat in their opinions of whether supervisors should be more experienced than supervisees in the specific activities and interventions they supervise. Although most interviewees emphasised the importance of recruiting experienced and knowledgeable supervisors, some noted that supervisors need not necessarily have direct experience of delivering the specific intervention type that the supervisee is carrying out.

For situations where a supervisor does not possess extensive technical expertise in the relevant area, Participant C suggested the following arrangement:

…*if you get a difficult case needing a lot of technical input you could then go to the person with the technical expertise and ask them and bring them in for a separate sort of consultation—“Can you come to my supervision session because my guy*’*s worrying about this case and we need your technical input”*.

(Technical Expert—Field)

A common issue raised by informants was the requirement for supervisors to understand and respect the culture of the setting where the work is being carried out. This requires awareness of political context, attitudes, social norms, taboos, local idioms and ways of describing mental health and expressing emotional or psychological distress. In the worst cases, a lack of such awareness could risk exacerbating clients’ emotional pain, as described by Participant D:

…*I have definitely seen examples where you might be working in an extremely conservative setting*, *where there might be a lot of sensitivity around gender and sexuality*, *and I have seen foreign MHPSS practitioners*, *through translation*, *starting to start talk about* ’*anal stages*’ *and* ’*Oedipus complexes*’ *and things that are just culturally quite*… *well first of all the translator is baffled as to how do they translate this in a way that is not going to cause embarrassment and confusion and*, *you know*, *the concepts just might not be culturally acceptable or appropriate*.

(Technical Expert—Field)

In introducing unfamiliar mental health concepts, interviewees suggested that supervisors should work to incorporate social and cultural norms and avoid overwhelming supervisees with too much specialised or culture-specific terminology at once. Supervision should be tailored to the context as far as possible, while also respectfully confronting stereotypes, stigma and harmful cultural practices.

#### Supervision should be a separate process to line management

Participants reported that in many cases, separating supervision from line management is difficult. When the same person covers these two functions, supervisees might feel under pressure to perform and meet managers’ performance-related expectations. Therefore, interviewees highlighted that ideally, line managers should not be assigned as supervisors. Where these roles are combined, the power imbalance between supervisees and their line manager may compromise supervisees’ capacity to share their difficulties and discuss mistakes. As summarised by Participant H: *“If you can*’*t be vulnerable in supervision*, *it*’*s not effective”*. Combining supervision and line management also increases the risk that supervision is used as a platform for managerial and human resource issues (e.g. meeting targets, deadlines or contractual agreements).

Various strategies for distinguishing the role of a supervisor from that of a manager were proposed. Some organisations allocate different managerial staff to the supervisory role or use external supervision, as explained by Participant A:

*Sometimes it*’*s very helpful to have someone external from the organisation do the supervision*… *when it is someone internal it is hard to shake that line management* / *dual roles hat*. *Staff will likely think*: *“you are supervising me*, *and it feels safe and supportive*, *but you could also fire me"*. *That dynamic is unhelpful in supervision*.

(Technical Expert—HQ)

External supervision, through an inter-agency system, was presented as a potentially important resource for organisations that have limited capacity to carry out MHPSS supervision. Remote supervision was presented by Participant U as a third option for clearly delineating line management from MHPSS supervision:

*I*’*m a huge proponent of remote supervision*. *I think it is absolutely feasible if done correctly and*, *in the world that we live in its going to become more and more necessary*…*And again*, *it goes back to my strong belief that your supervisor should not be your direct manager*… *remote supervision is one way to solve that problem*.

(Technical Expert—HQ)

#### Supervision should be structured and have clear objectives

Participants expressed a clear preference for supportive supervision carried out on a one-to-one basis. As Participant C explained:

*What I wanted from supervision was a one-to-one with somebody who would listen to what I was struggling with and help me work out a way to deal with [clients] and manage them and care for them*…

(Technical Expert—Field)

Aside from this specification, informants felt that supervision should have clearly stated goals, based on both supervisors’ and supervisees’ expectations. Participants expressed that the start of a session should be marked by the co-constructing of an agenda that allows space for supervisees to discuss challenges and present success stories. Another suggested agenda point was the opportunity for supervisees to practice skills, present scenarios, and for supervisors to leverage these scenarios to introduce/review concepts, skills, strategies or guidelines. In addition, participants suggested that supervisors should make time to provide competency-based feedback to supervisees on their progress, discuss the personal impact of the work on their wellbeing, and request feedback on their own performance as supervisors.

Although most participants had a preference for one-to-one supervision provision, several participants noted distinct benefits and challenges associated with other approaches, including group, live, peer and remote supervision. The insights specific to each of these four modalities differ somewhat from those relating to individual supervision, as discussed in turn in the following subsections:

*Group supervision*. Group supervision is commonly used within MHPSS as it is considered cost-effective, good for team relations, and allows supervisees to support and learn from each other’s experiences. Group supervision sessions using a faciliatory approach by the supervisor can foster learning among supervisees. Some organisations carry out individual supervision sessions with newly trained providers, before incorporating them into group supervision. Others deliver the two supervision formats concurrently.

Group formats may include elements of interactive and didactic group discussions, as well as role-plays. Alternatively, group supervision sessions may involve reviewing a specific case or activity and gathering the groups’ perspectives on that case. Group supervision formats were seen as potentially useful for a multi-disciplinary team. However, arrangements that require convening meetings of professionals across disciplines can become administratively burdensome for supervisors:

*Sometimes [group supervision] would be OK if they*’*re all working on the same case*, *because then you have the nice MDT perspective*. *But if they*’*re not*, *it can be very difficult to keep them engaged because they have very different roles and therefore they have different things they want to talk about*. *It*’*s important to pitch supervision at the right level*. *But what happened to me in [country] for example*, *is*, *we had activities in 20 different sites and in each of those sites we had psychologists*, *psychiatrists and social workers*. *And so then*, *trying to even get everyone together when everyone is spread throughout the country—that alone is almost impossible*. *So*, *you have to rely on Skype and then if you don*’*t speak the language and not everyone speaks English then that makes it incredibly challenging*. *Even just the scheduling makes it incredibly difficult*.

(Participant A, Technical Expert—HQ)

Although group supervision with groups from the same disciplines can be easier to arrange for supervisors and less resource-intensive for organisations than individual formats, group supervision arrangements can be frustrating for supervisees. As explained by Participant C:

*I wanted to talk about my cases*. *I wanted to be heard and I didn*’*t want to take an hour out to go and listen to somebody else*’*s*.

(Technical Expert—Field)

Therefore, while group supervision was seen as a useful format in some instances, participants felt it should be used in combination with individual supervision and not as a substitute.

*Live supervision*. This format allows supervisors to directly observe supervisees’ practice, identify whether they are integrating new concepts and skills, give personalised feedback, identify areas for discussion at group and individual supervision sessions, and detect issues related to supervisees’ care and well-being.

However, this type of supervision requires the beneficiary’s consent and is more difficult where the supervisor does not speak the local language, as it may be necessary to also include an interpreter within the session. Participants viewed live supervision as particularly valuable when training new providers or when developing a new skill or intervention. In these early stages, some supervisors may choose to co-facilitate the first MHPSS sessions with the supervisees and observe sessions on an *ad hoc* basis thereafter. This can function to build trust between supervisor and supervisee, as explained by Participant G:

*I think this for me is the more successful way*. *I think also the relationship that you build with the person when you do that is irreplaceable because there is like a trust link and they don*’*t see you like “oh*, *you are the boss*!*” But you come to just kind of check on them*, *but it*’*s more like they also see you working*.

(Technical Expert—HQ)

*Peer supervision*. Peer supervision, less commonly referred to as ‘intervision’, consists of two or more providers supporting and learning from one another without a supervisor being present in the session. Peers can share solutions and tools to address challenges, and pool experiences, including strategies to cope with the personal impact of conducting MHPSS work. As described by participant T:

*there is good evidence that learning from your peers is just as effective as being taught by more senior people*, *you know*, *particularly in the long term solving “I saw this patient*, *I did not know what to do” problem [*…*] rather than just traditional supervision*.

(Technical Expert—HQ)

Any issues that are not resolved during peer supervision may then be taken up for discussion within one-to-one sessions with a supervisor. Peer supervision was generally viewed by informants as cost-effective and practical where there is no supervisor in place, or where supervisors have limited access to programme sites. Notably, it was viewed as particularly useful to overcome the challenges of hierarchy and power dynamics inherent to other supervision formats. Like group supervision, peer supervision was seen as a complementary approach, ideally not to be used as an alternative to one-to-one or group sessions. Peer support practices (e.g., conversations during lunch time or through WhatsApp), are also fostered by some organisations as informal peer support structures.

*Remote supervision*. While participants had a preference for face-to-face supervision, some also highlighted the value of remote supervision, especially in contexts where access is difficult and supervisors are scarce. However, internet connectivity issues, working with interpreters, miscommunication, and time differences are all potential impediments to successful implementation of this approach. Accordingly, remote supervision requires more time than what would normally be reserved for face-to-face sessions due to potential technical issues and allowing time for interpretation, if required.

Where remote supervision is used for a prolonged period, participants advised that supervisors should meet supervisees face-to-face at least once and live supervision, before the remote supervision commences. Doing this can give the supervisor a sense of supervisees’ skillset and establish a basic degree of trust and rapport. Where the supervisor has not had the opportunity to familiarise themselves with the context and supervisees in this manner, remote supervision may be more limited in its effectiveness.

#### There should be higher frequency of supervision after trainings and regular throughout service provision

All participants spoke of the importance of regular supervision, although they varied in their assessments of what constitutes regular. Some advocated weekly or fortnightly sessions, while others felt that monthly sessions were sufficient. Participants suggested that the frequency of supervision should be greater when a supervisee is first recruited or has recently trained in a new skill or intervention, with training ideally followed by at least six months of close supervision. Participant A highlighted the importance of regular supervision to encourage consistent reflection among providers:

*Sometimes*, *you don*’*t know when you need to raise something*, *especially if you are not receiving regular supervision*. *You kind of look back in hindsight and you think I probably should have spoken to someone about that*.

(Technical Expert—HQ)

When regularly scheduled meetings are not possible, some participants felt that longer individual sessions (e.g., two or three hours, depending on the supervision format) could act as a substitute. Overall however, most participants agreed that while important to have scheduled regular meetings, the frequency and duration should ultimately be based on supervisees’ needs.

#### Supervisors should be aware of power dynamics and use them constructively

Some participants referenced the challenge of navigating power dynamics in a supervisor-supervisee relationship, which may arise from professional hierarchies as well as social factors such as gender, culture and race. Participant H expressed:

*I think any*…*important subculture aspect of someone*’*s identity is relevant in clinical supervision*, *sexual orientation*, *gender expression*. *Religion*, *race*, *ethnic background*, *political background*. *It*’*s all important*.

(Technical Expert—HQ)

Most described navigating these dynamics as an inevitable aspect of supervision and highlighted the risks associated with supervisors not bringing awareness of their position of power or using it inappropriately. As explained by Participant N:

*Because of my background*, *I can unintentionally impose Western views on the people that I work with*. *And I can unintentionally like replicate colonisation by imposing those views*…*Because I*’*m in the dynamic*, *I don*’*t always recognise when it*’*s happening so my supervisor can hold me accountable and make sure that I*’*m not like replicating those unhelpful and quite harmful dynamics from the past*.

(Technical Expert—Field)

To manage the influence of power imbalances, some participants advocated an open discussion relating to personal boundaries prior to establishing a supervisory relationship, and again once the relationship is established. As part of this process, supervisees should be informed that supervision is about communication and creating an empowering, non-judgemental and non-punitive space, where the supervisor is there to support and listen, rather than to oversee.

Participants acknowledged that discussions on the topic of power dynamics can be aversive, particularly for those holding the positions of power. As Participant N explained:

*Like talking about how being a female in [country] might affect a client*’*s experience of mental health*…*And then using that as a platform to talk about how gender might play out in the supervision relationships*. *But that*’*s like very hard*. *I have been here since [date] and we have just been able to touch it*. *We are not able to talk about it yet*… *It means asking men to reflect on their position of power in society and it*’*s asking women to reflect on their position of power if they*’*re a woman from the dominant culture*… *So that doesn*’*t come easily to people I think*. *Generally*… *people don*’*t reflect on this*.

(Technical Expert—Field)

It was noted that individuals at every professional level have biases, prejudices or culturally-influenced attitudes that may impact negatively upon practice, and it is therefore important for supervision to continue, regardless of level of seniority.

#### Supervisees should have an active role in supervision and be committed to their professional development

Informants noted that good supervision is a ‘two-way street’ and supervisees should play an active role to make the most of sessions. Specifically, participants expressed that supervisees should prepare in advance for sessions, reflecting on relevant topics, cases or difficulties. The ideal supervisee was described as curious, open, reflective, able to challenge themselves and recognise when they need support. As explained by Participant P:

…*a willingness to learn*, *open to building new skills*, *just an openness and*, *being able to*, *you know*, *recognise when they might need help with something*…*or even recognise that we have a client who has specific challenges and seeing it as*…*an opportunity for a learning experience rather than such a difficult case*…

(Technical Expert—HQ)

To make the most of supervision, supervisees should also be trained on how to present a case in a way that allows all the relevant information to be communicated without breaching confidentiality (e.g., use of pseudonyms, use of gender-neutral pronouns etc.).

### Objective 3: Identifying how supervision can be best incorporated into existing MHPSS programming

#### Train supervisors in supervision

Although most participants who had experience in providing supervision had not received formal training, instead learning how to carry out the role mostly from their own experience as supervisees, there was broad consensus that supervisors should receive training to develop their supervisory skills. This was seen as particularly important as many supervisors come to the supervisory role because they reach a certain level of seniority, not necessarily because they possess the skills of a competent supervisor.

Suggested topics for training included: defining and structuring supervision tools for adult learning, establishing supervisory alliance, reflective questioning, setting boundaries, building cultural sensitivity, monitoring supervisees’ professional development and well-being, identifying transference and countertransference, conflict resolution, coaching, facilitation and challenging bias and harmful practices. Participant S described such a training programme as a positive example of what such training should entail, followed by live supervision to observe the application of new skills:

*Our supervisors were first MHPSS workers so they have experienced the same job tasks*—*responsibilities and challenges as the workers they will supervise*. *To train them as supervisors*, *we talk about leadership skills*. *We talk about the skills they need to be sure their workers are able to do their jobs like time management and proper assessment*…*We have lots of discussion about the management of a team*. *How to run a team meeting*? *How to sit with workers and listen fully to understand what they are truly doing in their daily work*? *How to ask questions to hear what the workers are doing well and hear what they*’*re not doing well*? *We practice skills for how to confront*—*challenge workers who do not seem to be doing well and much more*.

(Technical Expert—HQ)

#### Consider the need to strike a balance between quality of services delivered and quantity of beneficiaries reached

Some participants reflected on a perceived tension between supervision and scalability, given the focus of the latter on increasing numbers of beneficiaries. Supervision, on the other hand, focuses on increasing quality, empowering supervisees, and building capacity. Participant G described the following situation:

…*now with [number] programmes right it is just very difficult to maintain a very close supervision as we had before and I told you we have grown up exponentially in the past 8 years*…*I would say 8 years ago or say 5 years ago when we had [fewer] programs it was a lot easier but now it*’*s really challenging and then we have decided*…*to limit the growth because I don*’*t want to risk the supervision and the support system that we have built*.

(Technical Expert—HQ)

#### Build supervision into existing organisational structures

For supervision to work, participants expressed that it must be integrated within organisational structures. In order to achieve this, participants specified that (1) donors and organisations need to be aware of the critical role supervision plays in ensuring high-quality MHPSS programming; (2) the necessary number of supervisors should be recruited prior to providing any MHPSS services or training, the maximum supervisor-supervisee ratio should be established, and the ways in which supervisory roles differ from and interact with managerial roles clearly communicated; (3) supervisors and supervisees must be allocated time to conduct and participate in supervision, particularly during emergencies; (4) resources for supervision must be provided (e.g., training, transportation, private space to conduct sessions); (5) supervisors need clear job descriptions, that are informed by assessment of supervisees’ needs; (6) there needs to be a raising of awareness among MHPSS staff and volunteers (including providers and potential supervisees) of the added value and purpose of supervision and; (7) supervision must be adequately reflected in organisations’ monitoring and evaluation processes and programme-specific results frameworks.

#### Include design, monitoring and evaluation indicators and supporting tools to monitor supervisee wellbeing, progress and impact as part of MHPSS programming

Some suggested indicators proposed by informants to measure the impact of supervision include: supervisees feeling more comfortable to support clients and colleagues, and having an increased sense of their own resilience and wellbeing. Participants agreed that ideally, supervisees should be able to confidentially offer feedback to supervisors, though this may not be feasible where teams are small. Some participants discussed a more informal feedback mechanism that can work alongside confidential mechanisms, whereby space at the end of the session is dedicated to discussing what is working well and less well with the supervision process. Some informants reported using checklists and scales to monitor supervisees’ progress through a series of predetermined benchmarks of competence. These are usually based on supervisors’ observation of the providers’ practice during live supervision sessions, and are considered important for new supervisors, as explained by Participant A:

*Supervision tools can be very useful*. *I used to use them a lot in [country]*. *I think it is nice because it also shows supervisees something more tangible*. *It is still subjective at the end of the day*, *but it looks a bit more objective and it shows the progress they have made compared to where they started*.

(Technical Expert—HQ)

Participant E reported working with checklists using various benchmarks of successful supervision pertaining to both supervisors and supervisees. Aside from treatment fidelity monitoring forms, Participant E listed the following tools to guide evaluation of supervisory processes:

*“We have forms for the providers and then we have our own forms about what constitutes competent supervision in terms of knowledge of technical skills in terms of their ability to provide clear and simple effective feedback with respect*. *Ability to*, *you know*, *increase incrementally their expectations*…*ability to also tend to the trainees*’ *level of distress and self-care*…*”*

(Academic)

However, some participants reported challenges associated with checklists and argued that supervisors may use them inconsistently, preferring instead to rely on their own observations, interactive evaluation activities, or informal discussion within sessions.

## Discussion

This study used qualitative semi-structured interviews to explore key informants’ experiences of supervision in MHPSS programming, with a view to developing an integrated model for supervision (IMS) in humanitarian settings.

The proposed definitions of supervision centred around the creation of a safe, open and collaborative space in which supervisees can grow and develop their professional skills with the support of an experienced professional. The results highlighted an under-prioritisation of supervision in MHPSS programming in humanitarian emergencies. This is particularly concerning given the emotionally demanding nature of the work itself, coupled with the fact of carrying it out in very challenging and stressful contexts [18, 19]. MHPSS providers in humanitarian emergency settings often work in unstable contexts where there are significant safety and security concerns, working long hours, often while separated from their social support networks [18]. They are commonly exposed to both primary and secondary trauma in the course of their work, and as such as at an elevated risk of mental health problems [19]. Best practices for supervision include supervisors facilitating supervisees’ learning in a non-hierarchical or authoritarian manner, incorporating awareness of the relevant cultures or contexts. Interviewees expressed a strong preference for regular, individual supervision, with increased intensity following trainings e.g. weekly or biweekly sessions that may decrease to monthly after the “minimal standards of quality” are achieved [22]. Such an approach is embodied by the apprenticeship model for training [23]. In this model, supervisees report regularly to supervisors for a period of up to a year post-training, and supervisors in turn may report to their own supervisors or to trainers, allowing ongoing learning and adaptation in response to challenges [24].

Solutions for where face-to-face individualised support is difficult to achieve include group or remote supervision. Evidence suggests that both of these formats are already widely used in humanitarian contexts. For example, analysis of 15 trials of psychological interventions delivered by lay providers in LMICs suggested that group supervision was the most common format (86.7%), supplemented in some cases with individual supervision [25]. Additionally, although all the trials analysed by Singla et al. [25] used face-to-face supervision, in many cases this was supplemented with telephone (46.7%), or Skype (40.0%) support.

Few informants discussed the use of technological solutions to supervision [26] during the interviews. Although some noted challenges associated with using technology for individualised remote supervision, use of such solutions may be necessary where safety risks such as pandemics, insecurity and other access difficulties associated with humanitarian environments prevent in-person meetings from taking place. In areas of armed conflict security, for example, conducting in-person supervision in groups may attract the attention of the armed groups and evoke suspicion [27]. Technology can also be used to facilitate live supervision, for example, whereby supervisors review and provide feedback on audio or video recordings of interventions being carried out.

Furthermore in relation to best practices for supervision, informants expressed that ideally, supervision should be separate to line management in order to reduce the power imbalance inherent in the managerial relationship. Supervisors should maintain awareness of this and any other power asymmetries that may manifest in the supervisory relationship. Additionally, supervisees should take an active role in supervision, coming prepared to be reflective and open to discussion.

To integrate supervision into MHPSS programming, participants felt that training should be provided to supervisors, rather than staff acquiring a supervisory role simply because of increased seniority. Organisational and donor awareness of this requirement is necessary to encourage change; although participants reported that this awareness is growing, it is still an under-prioritised area that can sometimes exist in conflict with scalability concerns. Supervision also needs to be integrated into monitoring and evaluation frameworks—both for organisations and for individual programmes—that measure the benefits of effective supervision and capture the resulting development of supervisees’ confidence and capacities. Achieving this in practice requires budgeting for supervision at proposal stage, including it as a core function in job descriptions of relevant personnel, and organisational commitment to ensuring that the time and space for supervision is protected. The potential gains of enhancing organisational support structures are significant: recent research has shown perceived organisational support to be an important determinant of mental health among humanitarian volunteers [18].

Existing research in humanitarian and low-resource settings has emphasised the central role of supervision in fostering skills of providers, maintaining intervention fidelity and sustaining service delivery [28–30]. This is also reflected in the present findings, which provide an initial evidential basis for the development of an IMS for MHPSS in humanitarian settings. The IMS will provide guidance to enable all organisations, regardless of their size or resource availability, to implement supportive supervision practices that can reduce harm to supervisees, build their capacity and ultimately enhance service quality. Low resource settings are the norm in MHPSS provision, but this should not prevent supervision from being carried out. In fact, providers working for organisations with fewer resources should perhaps be considered even more vulnerable due to a higher risk of an overwhelming caseload.

To address the problem of resource scarcity, a graded approach may be useful, whereby supervision at a minimum is provided in a group setting, remotely, or by peers, in cases where circumstances do not allow for individual one-to-one sessions. However, the data gathered during these interviews strongly suggests that one-to-one sessions, on at least a monthly basis or as agreed upon with the supervisee, should be the minimum standard that all organisations strive to achieve in the context of limited funding. Each of group, remote and peer supervision are affected by specific limitations, several of which were outlined by the interviewees, and so the present findings appear to suggest that ideally these should be used as complementary approaches to individualised support.

The present interview findings suggest gaps in guidance in certain areas that may usefully be addressed within the IMS. For example, several informants noted the challenge of adapting supervision practice to be culturally appropriate. Power imbalances arising from all types of social factors and marginalisation experiences are important to consider in therapeutic relationships generally [31]. However, the common practice of organisations employing foreign supervisors from western contexts to supervise MHPSS providers from communities affected by humanitarian emergencies may create a particularly elevated risk of humanitarian supervisory mechanisms inadvertently reinforcing existing inequalities or power imbalances in this way [32]. This appears to indicate a need for the development cultural adaptation guidelines for MHPSS supervision within the IMS. Research has identified that various factors (e.g., limited time and human resources, poor security and logistics) commonly hinder organisations’ capacity to conduct thorough cultural adaptation processes [33, 34]. It may be the case that clearer guidance on cultural adaptation could assist organisations with addressing some of these barriers.

The inclusion within the IMS of template tools or checklists that can be adapted for monitoring and evaluation of supervision may also be useful. Guidance for supervisees to help them make the most of sessions may enhance the quality of the interaction, and help all parties conceptualise supervision as a ‘two-way street’. In terms of monitoring and evaluation, the key informant interviews point towards a range of potentially suitable indicators for measuring success of supervision. These include: change in supervisees’ own appraisals of their capacity to deliver services, increases in their sense of wellbeing, decreased stress or burnout, and qualitative insights in relation to their satisfaction with supervisory sessions. Other metrics of success may include supervisors’ assessments of supervisees’ progress, as well as more quantitative metrics such as the regularity and number of sessions completed. Kohrt et al. [35] have also tested a scale where lay providers assess peers’ clinical competence, although some challenges with this approach were noted, such as socially desirable scoring. Assessing the extent to which the implementation of the supervision practices described here produces meaningful impact in relation to supervisee wellbeing and client outcomes remains an important component of the development of the IMS.

The IMS, currently under development, is intended to work towards a cultural shift in the delivery of MHPSS in humanitarian settings. The model will provide a blueprint that can be adapted to organisations of different sizes operating in different types of humanitarian contexts to strengthen their supervision structures, to improve the quality of their programming and better protect the wellbeing of their workers. The findings of the present study, combined with desk research on effective supportive supervision and further stakeholder consultation and piloting, will inform the development of the IMS. The final guidance will consist of four sections: Section one will describe basic principles of supportive supervision, outlining the different ways it can be delivered, as well as considerations for its format and content. Section two will be designed to guide organisational leadership to incorporate supervision into their operations and organisational culture. Section three will focus on MHPSS practitioners and developing their supervisory skills, while the final section will guide supervisees in making the most of the supervisory process as a source of personal development and support in their work.

### Limitations

English-speaking participants from western countries were disproportionately represented in the present study, and most were highly educated and formally qualified. The interviewer and the research team are also employed by institutions in western countries. This reflects an asymmetry in the humanitarian sector generally, whereby professionals from non-western countries are under-represented at senior levels and skills and qualifications gained in western contexts are often required for career advancement [36]. This fact may mean that interviewees’ interpretations of problems relating to supervision in some contexts was not fully informed by knowledge of local systems issues. This limitation is also particularly important to bear in mind with respect to the present study when interpreting themes such as those relating to power imbalances. The general finding that power imbalances are sensitive and difficult to navigate may have differed had there been a proportionate representation of all cadres, staff and volunteers involved in MHPSS supervision. This question, therefore, of how to properly address power differentials is one that should not be considered as exhaustively answered by the present study. We propose that this area in particular is one that requires further research and analysis. Interviews were conducted in English which limited non-English speakers’ participation and might have acted as a barrier for interviews who were not native English speakers. In addition, the COVID-19 pandemic may have affected participation of personnel that did not have Internet access or frontline MHPSS providers responding to the pandemic. Since the study was envisioned to inform the IMS, it did not explore in-depth the structural barriers that affect the MHPSS sector (e.g., resources, availability and accessibility to training). Further research is needed to explore these barriers as well as implementation strategies to address them.

### Conclusions and implications

Despite its limitations, the present work provides first steps towards an integrated model for MHPSS supervision in humanitarian contexts. There are also several important strengths in the Methods applied, such as different authors carrying out the coding from those who conducted the interviews, and the cross-checks and validations carried out on the analysis. These practices ensure that the present article thoroughly reflects the content of the interviews and minimises bias in reporting.

Overall, there was strong support among participants for the development of a unified framework or model encompassing a definition of supervision, goals and guidance for the supervisory process. Implementing meaningful supervision structures was understood as part of humanitarian organisations’ duty of care to their workers, ensuring that workers develop the resilience and emotional resources to cope with the stressors they encounter through the content and contexts of their work. The present study provides preliminary evidence that improved supervision in MHPSS in humanitarian settings may have potential to protect supervisees’ wellbeing, reduce turnover, enhance skill development and improve the fidelity of MHPSS interventions. Although further evidence is needed in relation to the extent to which enhanced supervision will impact on these outcomes in humanitarian settings and whether there will be an associated tangible impact on service user outcomes, existing evidence suggest that significant gains are possible [14, 15]. If this is the case, then improving MHPSS supervision is an important step towards achieving the Sustainable Development Goals, particularly those relating to improving health outcomes and reducing inequalities within and between countries.

## Supporting information

S1 File Qualitative interview guide. (DOCX)
