# Supplementary material for: Towards an integrated model for supervision for mental health and psychosocial support in humanitarian emergencies: A qualitative study
Source: PLoS One. 2021 Oct 6;16(10):e0256077. doi: 10.1371/journal.pone.0256077 (PMC8494373; doi:10.1371/journal.pone.0256077)
Supplement: S1 File — (DOCX) [file pone.0256077.s001.docx]

# **Supervision: The Missing Link**

# **Semi-structured Interview Protocol for Front Line Workers and Technical Specialists**

Introduce yourself

Introduction of the Missing Link and reason for the interview:

*Thank you for agreeing to be interviewed for ‘Supervision: The Missing Link project’. The interview will be semi-structured and will last approximately one hour. The interviews will be conducted by Dr. Byron Bitanihirwe, Research Fellow with Trinity Centre for Global Health (Trinity College Dublin) and Kelly McBride, MHPSS Technical Advisor with IFRC Reference Center for Psychosocial Support.*

*The purpose of this interview is to inform an Integrated Model for Supervision that provides guidance for supervision of staff and volunteers implementing MHPSS and protection interventions in emergencies as well as guidance across different agencies. Ultimately, the aim to improve MHPSS service delivery through strengthened supervision.*

*Our discussion will be recorded, and securely stored. Everything that is discussed will be confidential, and we will ensure that your ideas and opinions are de-identified prior to any use. You can decline to this interview at any point in time, or not answer any questions that you do not feel comfortable responding to.*

*Before we begin, do you have any questions?*

*Do you agree to participate in this interview and understand that your responses are being recorded and will be used to inform the ‘Supervision: The Missing Link’ project?*

| Name |  |
| --- | --- |
| Position |  |
| Organization |  |
| Educational Background |  |
| Contact information |  |

## **Background and Experience**

**For all:**

1. How would you define the role and position you currently hold?
2. How would you describe your experience in Mental Health and Psychosocial Support (regardless of what sector)? And your experience with supervision?
3. How would you define supervision in relation to MHPSS programming?
4. Does your organization see supervision as a priority?
   1. If a priority, do you know how it is supported (e.g., funded, human resources), and why your organization is prioritizing it?
   2. If not, what gaps exist and what do you think is preventing it from being a priority?
5. Are you aware whether there are clear policies in place regarding supervision within your organization? If so, can you tell me about it, or if not, do you know why?
   1. Are you aware of any specific guidance information/material/resources that your organization uses to inform their supervision practices.
6. To what extent have you found supervision to be important in your work?

## **Supervision in Relation to Mental Health and Psychosocial Support**

**For all:**

1. What is the role of a supervisor? What competencies are necessary to perform effectively in the capacity of a supervisor?
2. What is the role of the supervisee? What competencies are needed for a supervisee to effectively take part in supervision?
3. Is it necessary to have supervision for those supporting MHPSS at all levels of the IASC Intervention pyramid (*if participant is not familiar with intervention pyramid, briefly provide overview*)? Why or why not?
4. Are there any resources that you have found to be helpful/unhelpful in the past related to supervision for MHPSS?
5. Do you have a supervisor yourself?
   1. Who are they (someone from your organization, external?) What is their professional background?
   2. How does your organization support you in receiving this supervision?
   3. How often do you meet with your supervisor and in what format? What do you find to be most helpful/unhelpful about your sessions?
   4. If you don’t have a supervisor, who do you seek support from or consult with if challenges arise in supervision? Have you asked your organization to support you by providing supervision?
6. If you have a supervisor, how often do you typically meet with them and how long do sessions typically last?
   1. How are these sessions usually structured?
   2. What are the goals of the sessions?
7. What different types or modalities of supervision are you familiar with? (e.g., individual, group, peer, remote, case presentations)
   1. From your experience, what modality fits best in your work or have you found to be the most helpful or unhelpful?
   2. Are there any modalities you would like to have more information or guidance on?
8. Do the supervision sessions that take place include aspects of self-care and staff wellbeing? If yes, can you tell me about it / if no, why not?
9. What do you feel are the most valuable aspects/do you get most from supervision?
10. Are there things that could be done to improve supervision sessions to better support you and your work? If yes, what could be done to make the supervision sessions better or done differently to better support you?

**For those providing Supervision:**

1. What (if any) training (formal/non-formal) as a supervisor have you received? (how was it structured and what was the length of the training? Who was the training provided by?)
2. Did your organisation (past or present) provide you with training to support you as a supervisor, or did you enter the organization with previous experience?
3. What is the best way to prepare someone to become a supervisor, and to support them in their supervisory role?
   1. In your opinion, have you received adequate training and support as a supervisor? If yes, can you describe, if no, why not?
4. Are there opportunities for ongoing professional development to assist you as a supervisor?
   1. Is there a mentorship or peer support system within your organization?
   2. What types of training and support would you like to have to support you as a supervisor?
5. Are there resources you refer to as a supervisor?
6. What elements of supervision must be present for you to feel that supervision has been successful?
7. How do you know if supervision is not going well, and what do you do to address those challenges?
8. How do you evaluate progress made by your supervisee (*if already answered in question 8, skip*)
9. Can you tell me about the methods in place to provide feedback during supervision?
   1. Are there any forms or scales that you find beneficial to provide feedback to your supervisee? To evaluate the quality of interventions?
   2. Are there mechanisms in place for your supervisee to provide feedback to you about supervision? To evaluate the quality of interventions?
   3. How often do you provide feedback to your supervisee (formal and non-formal)?
10. Is there a system in place for you to document your supervision sessions?
11. What do you believe are the best practices available regarding MHPSS supervision?

**D. Additional Information**

1. Is there anything else you would like to add or that you think is important for me to know?
2. Are there any individuals who it would be beneficial for us to talk with to inform this project?

Thank you very much for your time. If you think that there is anything else you would like to add, please feel free to contact us. Please note that we may reach out to you in the future if we have any questions or need any clarification on the topics discussed during this interview. Do we have your permission to contact you in the future?
